# Fine mapping and candidate gene mining of major QTL *QSL.caas-6BL.1* for spike length in bread wheat (*Triticum aestivum* L.)

**DOI:** 10.3389/fpls.2025.1744596

**Published:** 2026-01-22

**Authors:** Xiaohan Xie, Xianghua Meng, Kangzhen Ren, Yutao Cui, Wenjiao Zhang, Dengan Xu, Wujun Ma, Xuehuan Dai

**Affiliations:** 1 College of Agronomy, Qingdao Agricultural University, Qingdao, China; 2 School of Agriculture, Murdoch University, Perth, WA, Australia

**Keywords:** Fine Mapping, QTL, RNA-seq, spike length, wheat

## Abstract

**Background:**

Spike length (SL) is a key agronomic determinant of wheat spike architecture and yield potential. This study focused on fine‑mapping a major SL‑regulating quantitative trait locus (QTL), QSL.caas‑6BL.1, previously identified in a Doumai (DM) × Shi 4185 population.

**Methods:**

High‑density molecular markers were developed and used to screen recombinant families derived from the cross. Genotype–phenotype co‑segregation analysis in advanced generations was employed to delineate the QTL region.

**Results:**

The QSL.caas‑6BL.1 locus was delimited to a 3 Mb physical interval on chromosome 6B (IWGSC RefSeq v2.1). Gene annotation and transcriptome analysis of young spikes identified 25 high‑confidence genes within this region. Among these, the NAC transcription factor gene TraesCS6B03G1211800 emerged as the prime candidate, being the only gene that combined significant differential expression between parental near‑isogenic lines with coding‑sequence variants leading to amino acid changes.

**Conclusion:**

Our work narrows a major QTL to a precise genomic interval and pinpoints a promising candidate gene, providing a valuable resource for understanding the genetic control of wheat spike development and for marker‑assisted breeding.

## 1 Introduction

Wheat (*Triticum aestivum* L.) is a globally crucial staple crop, supporting human nutrition while facing growing demands for higher yield and environmental adaptability amid changing climates (Curtis and Halford, 2014). Spike architecture is a core determinant of grain sink capacity and thus a key target for yield improvement (Ortez et al., 2022; Zhang et al., 2022; Shen et al., 2025). Among spike-related traits, SL is a core agronomic trait that tightly links to multiple yield-related and plant architecture traits, making it a key target for wheat genetic improvement (Ji et al., 2021a; Wang et al., 2023). Notably, SL is positively associated with spikelet number per spike (Jantasuriyarat et al., 2004), and an allele from W7984 confers pleiotropic effects on both SL and spike compactness—a trait closely intertwined with SL in previous studies (Schuler et al., 1994; Wu et al., 2014; Yao et al., 2019; Liu et al., 2022). Beyond spike architecture, SL is also correlated with plant biomass and harvest index: SL was positively associated with shoot biomass, straw biomass per plant, and grain yield (Moghaddam et al., 1997), while other study confirmed its positive correlations with aboveground biomass, harvest index, and grain yield through systematic evaluation of yield-related traits (Donmez et al., 2001). Additionally, SL has been documented to correlate with thousand kernel weight and grain yield per plant, further highlighting its comprehensive impact on yield formation (Schuler et al., 1994; Wu et al., 2014; Yao et al., 2019; Liu et al., 2022). Collectively, these consistent correlations between SL and key agronomic traits underscore the critical importance of identifying and validating SL-regulating genetic loci for advancing wheat yield improvement.

SL is a complex quantitative trait regulated by multiple genetic loci and environmental factors. Advances in molecular biology and quantitative genetics have enabled the mapping of numerous genes and QTLs governing SL in wheat (Faris et al., 2003; Simons et al., 2006; Wu et al., 2014; Huo et al., 2016; Dixon et al., 2018; Xie et al., 2018; Ji et al., 2021; Zhang et al., 2022, 2023; Ding et al., 2024; Li et al., 2024). Among these, several key loci have been functionally characterized or finely mapped. For example, at the gene level, the Q gene on chromosome 5A, which confers the free-threshing character, has also been closely associated with SL, plant height (PH), and spike compactness (Simons et al., 2006; Xie et al., 2018). The cloned *TaCol-B5*, encoding a CONSTANS-like protein, increases both the spikelet number per spike and SL, as well as the number of tillers, leading to an increase in field-based grain yield (Zhang et al., 2022). TaAIRP2-1B has been further validated as a direct regulator of wheat SL (Zhang et al., 2023). For QTLs, *qSl-2B* on chromosome 2B, which is a major stable SL QTL mapped to the 60.06–73.06 Mb region (Ding et al., 2024). *QSl.cib-5A* on chromosome 5A, which explains 7.88–26.6% of phenotypic variance explained (PVE), has been mapped (Ji et al., 2021); two major SL QTLs (*QSL.caas-4AS* and *QSL.caas-4AL.1*) on chromosome 4A, with PVEs of 4.5–12.3% and 6.8–11.9% respectively, have been identified (Gao et al., 2015). Additionally, a SL-associated QTL cluster on chromosome 5A has been reported, highlighting the concentrated distribution of SL-regulating genetic loci in specific genomic regions (Zhai et al., 2016). Although an increasing number of QTLs associated with SL have been identified over the last decades, few such QTLs have been fully studied, which has hampered the utilization of their favorable alleles in wheat breeding.

In the present study, we aimed to fine-map a wheat major QTL *QSL.caas-6BL.1* for SL, which was previously identified using a recombinant inbred line (RIL) population derived from the cross between DM and Shi 4185 (Li et al., 2018). To achieve this, we developed high-density markers, screened residual heterozygous lines (RHLs), and performed genotype-phenotype co-segregation analysis using advanced generation families. These integrated experimental strategies successfully narrowed the *QSL.caas-6BL.1* locus to a 3.3 Mb physical interval (708.2—711.5 Mb) on chromosome 6B. Within this delimited region, integrated gene annotation and transcriptome analysis identified the NAC transcription factor gene *TraesCS6B03G1211800* as a strong causal candidate. Furthermore, we developed a functional competitive allele-specific PCR (KASP) marker that is directly applicable for marker-assisted selection in wheat breeding. Collectively, these findings establish a solid foundation for the map-based cloning of *QSL.caas-6BL.1* and provided a foundation for the genetic improvement of wheat SL-related traits in the future and provided more molecular markers for molecular breeding programs that aimed to improve the yield potential of wheat.

## 2 Materials and methods

### 2.1 Plant materials and population development

The fine-mapping population was derived from a cross between two winter wheat lines: the maternal parent DM and the paternal parent Shi 4185. RIL population from this cross was previously established (Li et al., 2018; Xu et al., 2019). The paternal line Shi 4185, a cultivar released in Hebei Province characterized by smaller spikes, higher tillering capacity, and lower plant biomass; and the maternal line DM, which exhibits larger spikes, broader flag leaves, greater leaf area, and reduced tillering ability (Li et al., 2018; Xu et al., 2019). For this study, we utilized F_2_ seeds from the same cross, obtained from the germplasm bank of the National Wheat Improvement Center in June 2022.

### 2.2 Field trials and phenotypic evaluation

Field trials were conducted over three consecutive growing seasons (2022–2025) at two experimental locations: Boxin Agricultural Technology Co., Ltd. (Zibo, Shandong Province) and the Jiaozhou Demonstration Park (Qingdao, Shandong Province), China. In the 2022–2023 season, the population was planted in a double-row plot design (row length: 1.5 m) with two replicate blocks. Individual F_2_ plants were screened to identify those heterozygous at the target *QSL.caas-6BL.1* region. These RHLs were self-pollinated to generate F_3_ seeds. In the subsequent 2023–2024 and 2024–2025 seasons, progeny rows from the selected RHLs (F_4_ and F_5_ generations) were planted in a three-row plot design with two replicate blocks at each location. SL was measured as the length of the main spike from the base of the rachis to the tip of the terminal spikelet, excluding awns. For each plant, three primary spikes were measured, and the average value was recorded as the representative SL. Plants were individually tagged, and phenotypic data were collected prior to tissue sampling for DNA extraction. Genomic DNA was extracted from leaf tissue using a high-salt and low-pH precipitation method. All field management followed local standard agronomic practices.

### 2.3 Fine—mapping and genotyping

Marker development was based on the reference genome of Shi 4185 (Jiao et al., 2025). Whole-genome resequencing data of DM (unpublished data) were aligned to this reference and using Geneious Prime software (v2023.0.4), a 21 Mb target interval (700–721 Mb) on chromosome 6B (IWGSC RefSeq v2.1) was defined based on the initial mapping results (approximately 705 Mb). Polymorphic InDels and single-nucleotide polymorphisms (SNPs) were identified within this region. These variants were then used to develop InDel, CAPS, and KASP markers. Initial comparative genomic analysis between the parental lines enabled the development of seven polymorphic markers (3 InDel, 1 CAPS, 3 KASP) within the target interval. These markers were used to genotype an F_4_ population of 492 individuals to identify plants harboring recombination events within the region. Two representative recombinant lines (L1–L2) were selected, as their recombination breakpoints collectively spanned the entire initial 21 Mb target interval. These plants were self-pollinated to generate five distinct F_4_:_5_ families. To enhance mapping resolution, a subsequent round of refined genomic comparison was performed, leading to the development of four additional high-quality polymorphic markers (1 InDel, 2 CAPS, 1 KASP) within critical regions indicated by preliminary analysis. This expanded set of 11 markers was deployed for high-density genotyping of approximately 230 individuals derived from the five F_4_:_5_ families (L3–L7). By analyzing the segregation patterns within each family, the recombination breakpoints in their respective F_4_:_5_ progenies were precisely determined and mapped to intervals between flanking markers, enabling a significant reduction of the target QTL to a narrower physical interval.

### 2.4 RNA-seq and qRT-PCR

Based on phenotypic evaluation, the parental lines DM and Shi 4185 showed significant differences in young spike length at the stamen and pistil differentiation stage. To investigate the molecular basis, we selected a near-isogenic line (NIL-133-9) homozygous for the target *QSL.caas-6BL.1* region from the cross population. Young spikes were collected from NIL plants homozygous for either the DM or Shi 4185 allele at the same developmental stage. For each genotype, spikes were collected from three different, independently grown plants, constituting three biological replicates. Samples were immediately frozen in liquid nitrogen and stored at -80 °C. Total RNA was extracted using TRIzol reagent. RNA integrity was verified with an Agilent 2100 Bioanalyzer. Libraries were constructed and sequenced on an Illumina platform with a PE150 strategy. Raw reads were quality-trimmed and aligned to the Chinese Spring reference genome (IWGSC RefSeq v2.1). Gene expression levels were quantified as TPM. Differential expression analysis was performed using thresholds of |log_2_(fold change)| > 1 and adjusted *P*-value < 0.05. Total RNA for RNA sequencing was reverse transcribed using the Evo M-MLV Reverse Transcriptase Pre-mix Kit (AG). Gene-specific primers were designed via Primer-BLAST. qRT-PCR was performed on the QuantStudio 5 Real-Time Fluorescent Quantitative PCR System (Applied Biosystems) using ChamQ Blue Universal SYBR qPCR Master Mix (Vazyme). The wheat *EF1α* gene served as the internal control. Relative expression levels were calculated using the 2^(-ΔΔCt) method. Each sample underwent triplicate technical replicates.

### 2.5 Statistical and bioinformatics analysis

Phenotypic data comparisons between genotypes were performed using Student’s *t*-test. Gene Ontology (GO) and Kyoto Encyclopedia of Genes and Genomes (KEGG) enrichment analyses of differentially expressed genes (DEGs) were conducted using TBtools, with significance set at an adjusted *P*-value < 0.05. Homologous genes in rice (*Oryza sativa* Japonica Group) and maize (*Zea mays*) were identified using the Ensembl Plants database and cross-referenced with functional annotations from literature. All statistical analyses and visualizations were performed in Microsoft Excel, R (v4.3.2) or TBtools (v2.363; Chen et al., 2023).

## 3 Results

### 3.1 Phenotypic validation of the major spike length QTL *QSL.caas-6BL.1*

**Figure 1:**
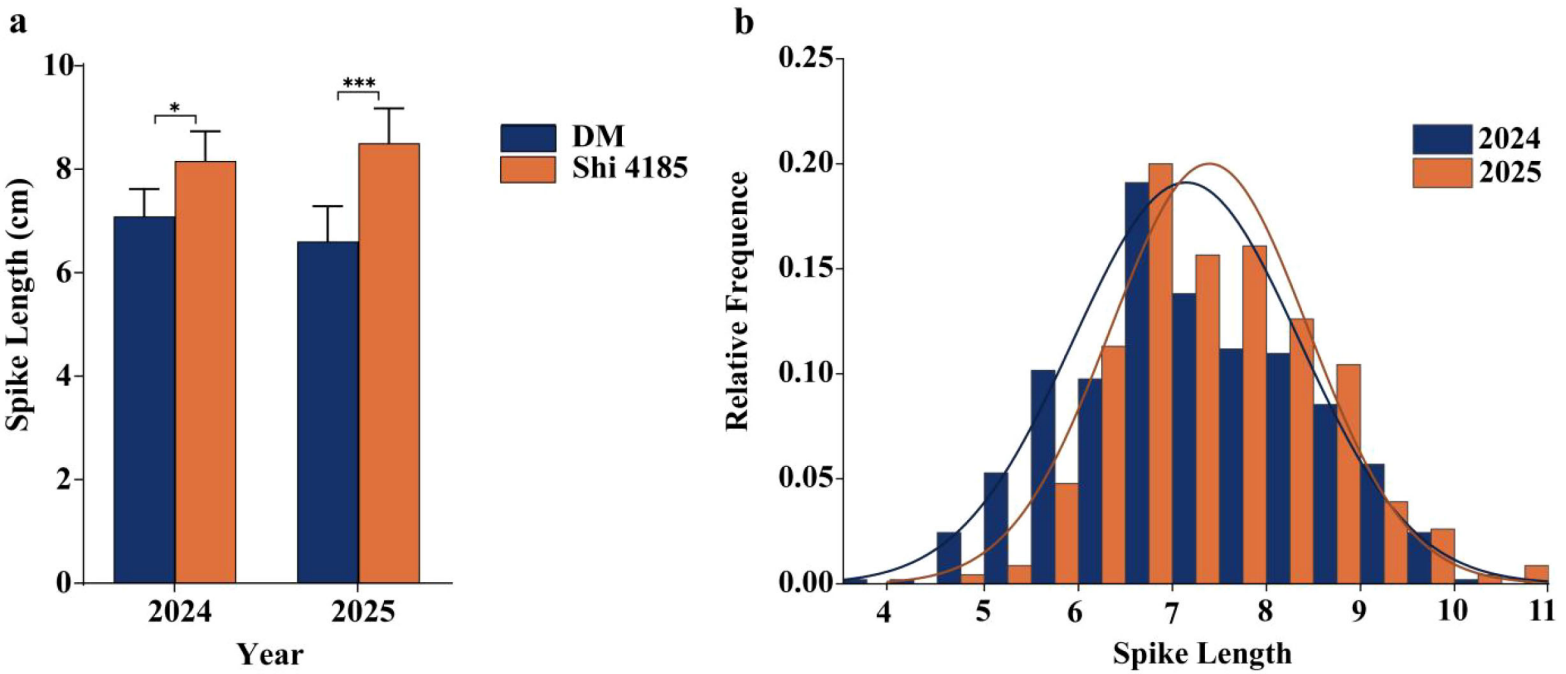
**(a)** Comparison of spike length between parental lines DM and Shi 4185. Bars represent mean ± standard deviation (n=10 plants per line). Statistical significance was determined by an unpaired *t*-test with Welch’s correction (*P < 0.05; ***P < 0.001). **(b)** Distribution of SL in the RIL population derived from DM × Shi 4185 across the 2023–2024 (Zibo, Shandong Province) and 2024–2025 (Qingdao, Shandong Province) experiments. Phenotypic values of the two parents were marked by vertical arrows.

To validate the major SL QTL *QSL.caas-6BL.1*, phenotypic characterization of the two parental lines (DM; Shi 4185) and the Residual heterozygous lines (RHLs) population (n=275) was conducted across two distinct environments (Zibo, Shandong Province in 2024; Qingdao, Shandong Province in 2025). The parental lines exhibited significant and consistent differences in SL (*P* < 0.05) across all tested environments. DM had an average SL of 7.09 ± 0.74 cm in 2024 and 6.60 ± 1.07 cm in 2025, while Shi 4185 had longer spikes with an average of 8.16 ± 0.96 cm in 2024 and 8.51 ± 0.82 cm in 2025 (**Figure 1a**; **Supplementary Table 1**). This consistent phenotypic difference confirms that Shi 4185 contributes the allele for increased spike length. In the RIL population, SL displayed continuous variation over a wide range: specifically, SL ranged from 3.80–10.50 cm in 2024 and 4.50–11.50 cm in 2025. The frequency distributions of SL in the population approximated normal distributions across both environments (**Figure 1b**; **Supplementary Table 1**). These results confirm that wheat SL is a quantitative trait controlled by multiple genes and validate the suitability of this population for subsequent QTL mapping of SL.

### 3.2 Fine mapping of the SL-regulating QTL *QSL.caas-6BL.1*

To finely map the major SL-regulating QTL *QSL.caas-6BL.1*, we identified kompetitive allelespecific PCR (KASP) markers on chromosome 6BL between DM and Shi 4185. Seven molecular markers (*INDEL691*, *CAPS699*, *INDEL702*, *INDEL704*, *KASP704*, *KASP705*, *KASP711*) were mapped into the genetic region of *QSL.caas-6BL.1* (**Supplementary Figure 1**; **Supplementary Table 2**). Using these markers, we screened recombinants from the F_4_ population (492 individuals) of the cross DM/Shi 4185. This screening identified 180 recombinant individuals carrying crossovers within the target interval (700–721 Mb) (**Supplementary Figure 1**, **Supplementary Table 2**). Within these, two key recombinant individuals (L1–L2) were selected for further analysis—their recombination breakpoints collectively spanned the entire 700–721 Mb region, and their progeny (F_5_ lines) derived via self-pollinated were used for subsequent high-resolution mapping.

**Figure 2:**
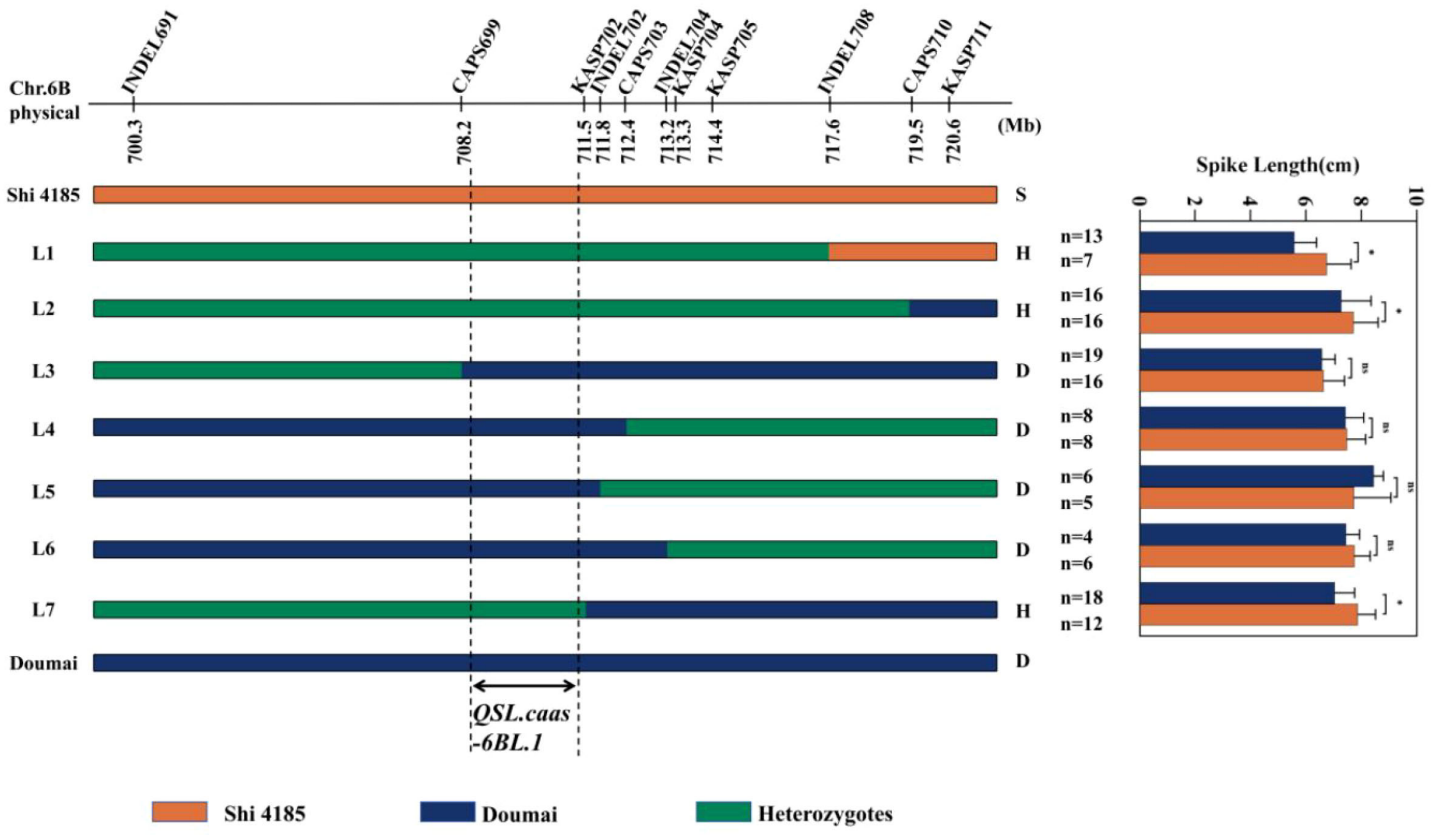
Fine mapping of SL-regulating QTL *QSL.caas-6BL.1* based on F_4_:_5_ recombinant families. Recombinants L1–L2 correspond to F_4_ plants (2023–2024), while L3–L7 correspond to F_5_ plants (2024–2025). Genotypic patterns are indicated by colors: dark blue and “D” represent homozygous DM alleles; orange and “S” represent homozygous Shi 4185 alleles; green and “H” represent heterozygous genotypes. Recombination breakpoints (vertical dashed lines) are positioned between adjacent markers based on genotyping data. The right panel shows statistical comparison of spike length between DM-homozygous and Shi 4185-homozygous progeny derived from self-pollinated heterozygous recombinants. Asterisks (*) indicate significant differences (*P* < 0.05, Student’s *t*-test); “*ns*” indicates non-significant differences.

In the subsequent growing season, five F_5_ families (L3–L7) were genotyped to precisely determine the recombination breakpoints of their corresponding F_4_ parent individuals. To refine genotyping resolution, four additional markers (*KASP702*, *CAPS703*, *INDEL708*, *CAPS710*) were developed for critical regions identified in the initial mapping, establishing a high-density genotyping panel of 11 markers (**Supplementary Figure 1**; **Supplementary Table 2**). By analyzing segregation patterns within each family, the high-density genotyping data enabled precise mapping of recombination breakpoints as intervals between adjacent markers in these nine critical F_4_:_5_ recombinant lines (L1–L7). **Figure 2** illustrates the distribution of these recombination breakpoints, which are positioned between markers and partition the target interval into multiple sub-intervals. Through phenotypic evaluation and genotype-phenotype co-segregation analysis of the F_4_:_5_ families, the causal genetic interval responsible for spike length variation was ultimately delineated to a 3.3 Mb physical region (708.2–711.5 Mb) (**Figure 2**).

The *QSL.caas-6BL.1* locus was initially identified within a genomic region on chromosome 6BL known to harbor a cluster of QTLs for multiple yield-related traits. In the primary QTL analysis of the same DM × Shi 4185 population, Li et al. (2018) reported that the interval containing *QSL.caas-6BL.1* co-localized with stable QTLs for thousand-kernel weight (*QTKW.caas-6BL*), spike dry weight (*QSDW.caas-6BL*), heading date (*QHD.caas-6BL*), and plant height (*QPH.caas-6BL*). This preliminary evidence suggested that the genetic region responsible for spike length might also influence other key components of grain yield, providing the impetus for the present fine-mapping study.

### 3.3 Genomic characterization of the target region

Following the fine-mapping of the major SL-regulating QTL *QSL.caas-6BL.1* to a 3.3 Mb physical interval (708.2–711.5 Mb), we performed an integrated analysis of this genomic region. Based on the IWGSC RefSeq v2.1 genome annotation, 25 high-confidence genes within this interval were identified (**Table 1**). To explore genetic variations underlying SL differences between parents, we conducted comparative genomic analysis using genome assemblies of DM (unpublished data) and Shi 4185 (Jiao et al., 2025). This analysis revealed sequence variations in the open reading frames (ORFs) of 18 genes between the two parents, consisting of missense mutations derived from SNPs in 10 genes and frameshift or disruptive mutations caused by insertions/deletions (InDels) in 14 genes (detailed in **Supplementary Table 3**). To prioritize the most promising candidate genes for SL regulation, we conducted a systematic screening of sequence variations between DM and Shi 4185. This screening identified three genes—*TraesCS6B03G1207300*, *TraesCS6B03G1211400*, and *TraesCS6B03G1211800*—as strong candidates potentially directly involved in SL development based on the presence of protein-altering mutations. The remaining 22 genes were excluded from further consideration, as they either lacked functionally meaningful sequence variations or had no documented association with known spike development regulatory pathways.

**Table 1:** List of 25 genes within the interval of the fine-mapped QTL *QSL.caas-6BL.1*.

Gene stable ID	Gene start (bp)	Gene end (bp)	Description
*TraesCS6B03G1206600*	708240247	708246484	Acetyl-coenzyme A synthetase, chloroplastic/glyoxysomal
*TraesCS6B03G1206700*	708254593	708255462	Ethylene-responsive transcription factor ERN1
*TraesCS6B03G1206800*	708256992	708258122	NA
*TraesCS6B03G1206900*	708264316	708267523	Cold-responsive protein kinase 1
*TraesCS6B03G1207200*	708422475	708436062	Myosin-8
*TraesCS6B03G1207300*	708437256	708442632	Protein CHROMATIN REMODELING 4
*TraesCS6B03G1207500*	708774606	708779664	DNA binding protein, putative
*TraesCS6B03G1208900*	710179756	710180085	Ripening-related protein
*TraesCS6B03G1209300*	710370922	710375256	NBS-LRR resistance-like protein
*TraesCS6B03G1209700*	710602960	710604687	Protein PLASTID MOVEMENT IMPAIRED 1
*TraesCS6B03G1209800*	710607740	710613728	Actin-related protein 2/3 complex subunit
*TraesCS6B03G1210000*	710687817	710704284	RING finger and transmembrane domain-containing protein 2
*TraesCS6B03G1210300*	710872091	710878269	Cytochrome P450, putative
*TraesCS6B03G1210400*	710877505	710879495	Inosine-5’-monophosphate dehydrogenase
*TraesCS6B03G1210900*	710885488	710888757	Potassium-transporting ATPase potassium-binding subunit
*TraesCS6B03G1211000*	711079219	711083821	Telomere-binding family protein
*TraesCS6B03G1211100*	711087751	711091357	3-ketoacyl-CoA thiolase-like protein
*TraesCS6B03G1211200*	711103345	711107747	50S ribosomal protein L13
*TraesCS6B03G1211400*	711191672	711194064	Auxin-responsive protein IAA10
*TraesCS6B03G1211500*	711194279	711198838	Potassium channel beta subunit
*TraesCS6B03G1211700*	711199729	711205421	Riboflavin biosynthesis protein PYRR, chloroplastic
*TraesCS6B03G1211800*	711359763	711360320	NAC domain protein
*TraesCS6B03G1213400*	711459089	711460891	Pentatricopeptide repeat-containing protein At2g29760, chloroplastic
*TraesCS6B03G1213600*	711461821	711463051	Heavy metal transport/detoxification superfamily protein
*TraesCS6B03G1213800*	711521175	711522070	CTC-interacting domain 4

### 3.4 Transcriptome analysis of candidate genes in the *QSL.caas-6BL.1* Interval

**Figure 3:**
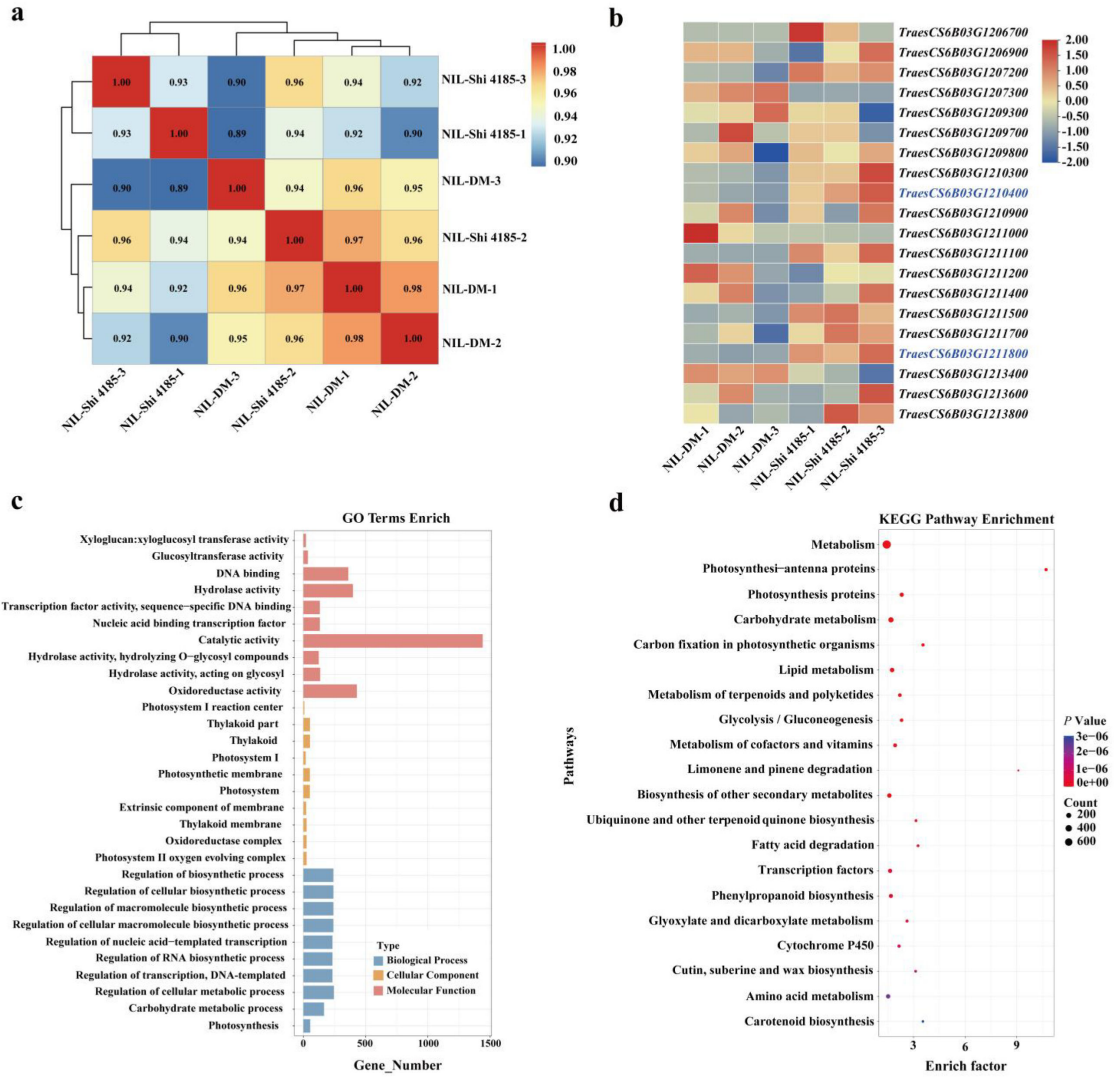
Expression analysis and functional enrichment of candidate genes based on RNA-Seq. **(a)** Sample correlation heatmap. Samples correspond to young spikes at the stamen-pistil differentiation stage from DM and Shi 4185 near-isogenic lines (NILs) (three biological replicates per genotype). The color scale represents Pearson correlation coefficients (r), ranging from deep blue (r=-1, perfect negative correlation) to white (r=0, no correlation) to bright red (r=+1, perfect positive correlation). Darker red hues indicate higher positive correlations between sample transcriptomes. **(b)** Heatmap of candidate gene expression. Candidate genes are derived from the 3.3 Mb fine-mapped interval of *QSL.caas-6BL.1*. Expression levels (TPM) in young spikes of DM and Shi 4185 NILs at the stamen-pistil differentiation stage are presented as row-wise Z-scores. The color scale ranges from blue (Z-score≤-2, significantly down-regulated relative to the gene’s mean) to white (Z-score=0, mean expression) to red (Z-score≥+2, significantly up-regulated relative to the gene’s mean). This visualization highlights expression patterns across samples for each gene. **(c)** Gene Ontology (GO) enrichment analysis. Significantly enriched terms in Biological Process, Cellular Component, and Molecular Function categories are shown. Enriched processes include DNA/nucleic acid binding, transcriptional regulation, chromosome organization, and cell cycle. **(d)** Kyoto Encyclopedia of Genes and Genomes (KEGG) pathway enrichment. Bubble size represents the number of differentially expressed genes (DEGs) enriched in each pathway, and color intensity indicates enrichment significance. Key enriched pathways include chromosome-associated proteins and DNA replication proteins, supporting roles in chromatin structure and cell cycle regulation.

To further precisely identify candidate genes regulating SL within the 3.3 Mb *QSL.caas-6BL.1* interval, we performed RNA-seq analysis on young spikes of DM and Shi 4185 near-isogenic lines at the stamen and pistil differentiation stage. Three biological replicates were included for each genotype to ensure data reliability. Principal component analysis (PCA) of the transcriptomic data showed that replicates from the same parent clustered tightly, while clear separation was observed between the two parental genotypes (**Supplementary Figure 2**). This result indicated that genotype was the primary driver of transcriptomic variation. Additionally, a kinship matrix heatmap constructed from genome-wide expression data confirmed high intra-group reproducibility, further validating the rigor of the experimental design and the quality of the RNA-seq data (**Figure 3a**).

Subsequent differential expression analysis identified 5,046 differentially expressed genes (DEGs) (|log_2_FoldChange| > 1 and *P*-adjust < 0.05) when mapped to the Chinese Spring reference genome (IWGSC RefSeq v2.1) (**Supplementary Table 4**). We then focused specifically on the 25 high-confidence genes within the 3.3 Mb fine-mapped *QSL.caas-6BL.1* interval. Among these, 23 genes showed no significant differential expression between DM and Shi 4185 NILs (**Figure 3b**; **Supplementary Tables 3**–**5**). Of the remaining two DEGs (*TraesCS6B03G1210400* and *TraesCS6B03G1211800*), although *TraesCS6B03G1210400* shows differential expression, comparative genomic analysis (as shown in **Supplementary Table 3**) revealed no non-synonymous SNPs or insertion-deletion (InDel) mutations within its coding sequence. Therefore, *TraesCS6B03G1211800* was the gene that exhibited both significant differential expression between parents and coding sequence variations (**Figure 3b**; **Supplementary Tables 3**–**5**), highlighting it as a high-priority candidate gene.

To characterize their functional roles, we performed Gene Ontology (GO) and Kyoto Encyclopedia of Genes and Genomes (KEGG) enrichment analyses on 5,046 DEGs (**Figures 3c, d**; **Supplementary Tables 6**, **7**). For GO enrichment, the top 10 enriched terms in each of the three core categories—cellular component, biological process, and molecular function—were visualized. Among these, the molecular function category, “catalytic activity (GO:0003824)” was the most prominently represented term, reflecting the involvement of metabolic enzyme activities in spike development. In the cellular component category, terms related to photosynthetic structures—including “thylakoid part (GO:0044436)”, “thylakoid (GO:0009579)”, “photosynthetic membrane (GO:0034357)”, and “photosystem (GO:0009521)”—were significantly enriched, consistent with the critical role of spike photosynthesis in grain development. In the biological processes category, enriched terms were mainly associated with regulatory processes, such as “regulation of DNA-templated transcription (GO:0006355)” and “regulation of cellular biosynthetic process (GO:0031323)”, indicating that transcriptional regulation is a key mechanism underlying SL variation (**Figure 3c**; **Supplementary Table 6**). KEGG pathway analysis further revealed the top 20 significantly enriched pathways. These DEGs were mainly associated with metabolic pathways, including carbohydrate metabolism, biosynthesis of secondary metabolites, and amino acid metabolism (**Figure 3d**; **Supplementary Table 7**). These results align with the dynamic physiological and biochemical changes occurring during young spike development, providing a functional framework for understanding the molecular mechanisms of SL regulation mediated by *QSL.caas-6BL.1.*

To independently validate the reliability of our transcriptome data, we performed quantitative real-time PCR (qRT-PCR) analysis on six selected genes using the same RNA samples employed for RNA-seq. The gene set included two DEGs from the fine-mapped *QSL.caas-6BL.1* interval (*TraesCS6B03G1211800* as the prime candidate and *TraesCS6B03G1210400*) and four additional DEGs randomly picked from the genome-wide RNA-seq dataset for expanded validation. The qRT-PCR results showed a highly significant positive correlation with the RNA-seq data (**Supplementary Figure 3**; **Supplementary Table 2**).

### 3.5 Integrated multi-evidence analysis identifies *TraesCS6B03G1211800* as the causal candidate

To robustly prioritize the causal gene from the 25 candidates and mitigate the risk of false negatives from expression screening alone, we employed a multi-layered evidence integration strategy. Homology and functional domain analysis indicated several genes with plausible roles in development (**Supplementary Figure 4**). Notably, *TraesCS6B03G1211800* encodes a NAC domain transcription factor, a family key to meristem regulation. Its rice ortholog (*Os07g0225300)* belongs to a clade containing known panicle architecture regulators (Anjum and Maiti, 2024). Furthermore, protein sequence and domain comparison with a curated set of cloned spike development genes in wheat, rice, and maize (**Supplementary Table 8**) revealed that the NAC domain of *TraesCS6B03G1211800* shares high similarity with those of established regulators such as *OsNAC121* (*Os10g0571600*) (Anjum and Maiti, 2024).

Comparative analysis with previously reported spike-related QTLs/genes confirmed the functional relevance of this genomic region on chromosome 6BL (Simons et al., 2006; Dixon et al., 2018; Du et al., 2020; Kuzay et al., 2022; Liu et al., 2022; Anjum and Maiti, 2024; Lin et al., 2024; Agata, 2025). This interval overlaps with a yield-QTL cluster on 6BL, which contains *TraesCS6B03G1211100* (*KAT-2B*)—a validated grain weight regulator—further supporting the functional importance of this chromosomal segment. However, genetic co-segregation analysis in our key recombinant families showed that the spike length phenotype perfectly tracked the haplotype of *TraesCS6B03G1211800*, but not that of *TraesCS6B03G1211100*, genetically separating the SL QTL from the linked grain weight effect. Conclusive integration of molecular evidence pinpointed the final candidate. *TraesCS6B03G1211800* was the only gene within the fine-mapped interval that satisfied all three decisive criteria: 1) significant differential expression in young spikes, 2) presence of a protein-altering missense mutation, and 3) genotype-phenotype co-segregation. Therefore, through systematic integration of cross-species homology, genetic dissection, and transcriptomic-genomic variation, we conclusively identify *TraesCS6B03G1211800* as the prime candidate gene underlying the *QSL.caas-6BL.1* QTL for spike length.

## 4 Discussion

SL is a key agronomic trait shaping wheat yield, and identifying major SL QTLs facilitates targeted crop improvement. This study fine-mapped the major SL QTL *QSL.caas-6BL.1* to a 3.3 Mb physical interval (708.2–711.5 Mb) on chromosome 6B via high-density genetic mapping and multi-environment phenotypic evaluations (**Figure 2**). Comparative analysis with previously reported loci revealed its genomic significance: *QSL.caas-6BL.1* is physically proximal but non-overlapping with the spike compactness QTL *QSc.cau-6B.1* (710.83–712.49 Mb; (Zhu et al., 2025). This adjacent distribution suggests the distal 6BL region is a genomic hotspot harboring independent loci that coordinately regulate spike architecture (Salina et al., 2022). Notably, *QSL.caas-6BL.1* exhibits consistent genetic effects across multiple environments (**Figure 1**; **Supplementary Table 1**), distinguishing it from many environmentally unstable SL QTLs reported previously (Bonneau et al., 2013; Sukumaran et al., 2018). The fine-mapping strategy, leveraging large-scale F_4:5_ populations to resolve recombination breakpoints, effectively narrowed the initial ~705 Mb region to 3.3 Mb—overcoming the limitation of excessively large target intervals (Raj and Nadarajah, 2022). Functional markers developed for *QSL.caas-6BL.1* (**Supplementary Figure 1**) enable direct application in marker-assisted selection, accelerating the development of long-spike wheat germplasm. Collectively, these features make *QSL.caas-6BL.1* a valuable locus for synergistic improvement of spike traits via gene pyramiding in breeding programs.

To identify causal genes from 25 high-confidence genes in the *QSL.caas-6BL.1* interval, a multi-evidence integration framework was established. This framework systematically integrated genetic (recombination breakpoint mapping), genomic (parent-specific sequence variation), transcriptomic (stage-specific expression), and comparative genomic data—reducing the candidate pool and enhancing inference reliability. Transcriptome analysis of young spikes at the stamen-pistil differentiation stage (a critical SL-determining stage) provided key functional evidence. *TraesCS6B03G1211800* was the only gene showing both significant differential expression between parents and coding sequence variation (**Figure 3b**; **Supplementary Tables 3**–**5**). It was significantly up-regulated in the long-spike parent Shi 4185 relative to DM (|log_2_FoldChange| > 1, *P* < 0.05), with expression timing coinciding with the critical SL-determining window. This positive correlation strongly supports it as a positive regulator of spike development. To address the inherent challenge of candidate selection within a 3.3 Mb interval, we moved beyond single-omics filtering. Our integrated framework synthesized cross-species homology (linking *TraesCS6B03G1211800* to known NAC regulators), direct comparison with cloned spike genes, and critical genetic co-segregation data that excluded the physically linked *KAT-2B* grain weight gene. This multi-evidence convergence uniquely singled out *TraesCS6B03G1211800*, as it alone combined differential expression, coding sequence polymorphism, and genetic linkage with the trait. This rigorous approach minimizes false-negative risk and provides a high-confidence target for functional validation of *QSL.caas-6BL.1*.

*TraesCS6B03G1211800*, as a NAC transcription factor-encoding gene, belongs to a family well-documented to govern plant organ morphogenesis (Xiong et al., 2025). Three potential molecular mechanisms are hypothesized: 1) acting as a transcriptional activator to up-regulate cell cycle-related genes (e.g., Cyclins) in rachis meristems, prolonging meristem activity and promoting cell proliferation (Zhang et al., 2021); 2) repressing boundary-specifying genes (e.g., CUC family) to delay rachis termination and extend elongation (Zhao et al., 2023); 3) integrating auxin and cytokinin signaling to establish a hormonal balance favoring rachis cell elongation (Nolan et al., 2020). Promoter region sequence variations (identified in genomic comparisons) may explain its up-regulation in Shi 4185, providing a target for subsequent validation (e.g., CRISPR/Cas9-mediated editing or promoter-reporter assays).

Interestingly, our fine-mapped interval for *QSL.caas-6BL.1* contains *TraesCS6B03G1211100*, which encodes the keto-acyl thiolase *KAT-2B*, a recently reported regulator of grain weight and yield in tetraploid wheat (Chen et al., 2020). Although this physical overlap suggests *KAT-2B* could underlie our QTL, our data argue against this. First, *TraesCS6B03G1211100* showed no significant differential expression in young spikes at the critical developmental stage. More decisively, co-segregation analysis in the key recombinant families revealed individuals in which the *KAT-2B* haplotype did not predict spike length, genetically dissociating it from the *QSL.caas-6BL.1* effect. We therefore propose that the 6BL 708.2–711.5 Mb region is a multi-trait regulatory hotspot: *KAT-2B* primarily modulates grain filling, while a distinct element—with *TraesCS6B03G1211800* as the lead candidate—controls spike elongation. This model of closely linked, functionally independent genes fits the noted clustering of spike morphology QTLs in this genomic region.

Our fine-mapping of *QSL.caas-6BL.1* refines a locus that was initially linked to a suite of yield-related traits, including thousand-kernel weight and spike dry weight (Li et al., 2018). The co-localization of these QTLs in the same genetic background presents a classic scenario of either pleiotropy or tight linkage. By delimiting the core physical interval for spike length to 3.3 Mb, this study provides a critical resource to distinguish between these possibilities. The presence of distinct candidate genes within this interval—such as the NAC transcription factor *TraesCS6B03G1211800* for spike elongation and the keto-acyl thiolase *KAT-2B* (Chen et al., 2020) implicated in grain weight—favors the model of a tightly linked gene cluster. This genetic architecture is highly relevant for breeding: it enables marker-assisted selection to pyramid favorable alleles for multiple traits, while also allowing for the selection of recombinants to break undesirable linkages. Future work employing near-isogenic lines for the fine-mapped segment will be essential to conclusively dissect its effects on individual yield components and to fully exploit its potential in wheat improvement.

GO and KEGG enrichment analyses of differentially expressed genes (DEGs) the QTL *QSL.caas-6BL.1* interval further illuminated SL regulation (**Figures 3c, d**; **Supplementary Tables 6**, **7**). DEGs were predominantly associated with catalytic activities, carbohydrate/amino acid metabolism (supporting energy and material demands for rachis cell proliferation/elongation; (Ryall et al., 2015), chloroplast-related components indicating photosynthetic support for spike growth; (Loudya et al., 2021), and transcriptional regulation (governing meristem activity and rachis elongation; (Scacchi et al., 2010; Wang et al., 2018). These results demonstrate *QSL.caas-6BL.1* influences SL through integrated transcriptional regulation and metabolic support (Lin et al., 2024; Liu et al., 2025). The multi-omics integration framework developed here provides a valuable template for dissecting complex traits in wheat and other polyploid crops (Yang et al., 2021). Future research will focus on validating the biological function of *TraesCS6B03G1211800*, elucidating its molecular mechanisms, and investigating its interactions with other spike-related QTLs to clarify the coordinated regulatory network of wheat spike architecture (Ai et al., 2024).

## 5 Conclusions

This study successfully fine-mapped the major SL-regulating QTL *QSL.caas-6BL.1* to a 3.3 Mb physical interval on chromosome 6B in wheat. Using an integrated multi-omics approach, we developed an effective gene identification strategy and identified the NAC transcription factor gene *TraesCS6B03G1211800* as the key candidate gene. Its significant upregulation in the long-spike parent suggests it regulates spike length through meristem development. The functional markers developed here provide practical tools for breeding, while our multi-omics framework offers a valuable paradigm for complex trait dissection in polyploid crops. Future work will focus on functional validation using gene editing and elucidating its regulatory network.

## Data Availability

The datasets presented in this study can be found in online repositories. The names of the repository/repositories and accession number(s) can be found in the article/**Supplementary Material**.
