# Supplementary material for: Fine mapping and candidate gene mining of major QTL QSL.caas-6BL.1 for spike length in bread wheat (Triticum aestivum L.)
Source: Front Plant Sci. 2026 Jan 22;16:1744596. doi: 10.3389/fpls.2025.1744596 (PMC12872863; doi:10.3389/fpls.2025.1744596)
Supplement: Supplementary file 1 [file Image1.pdf]

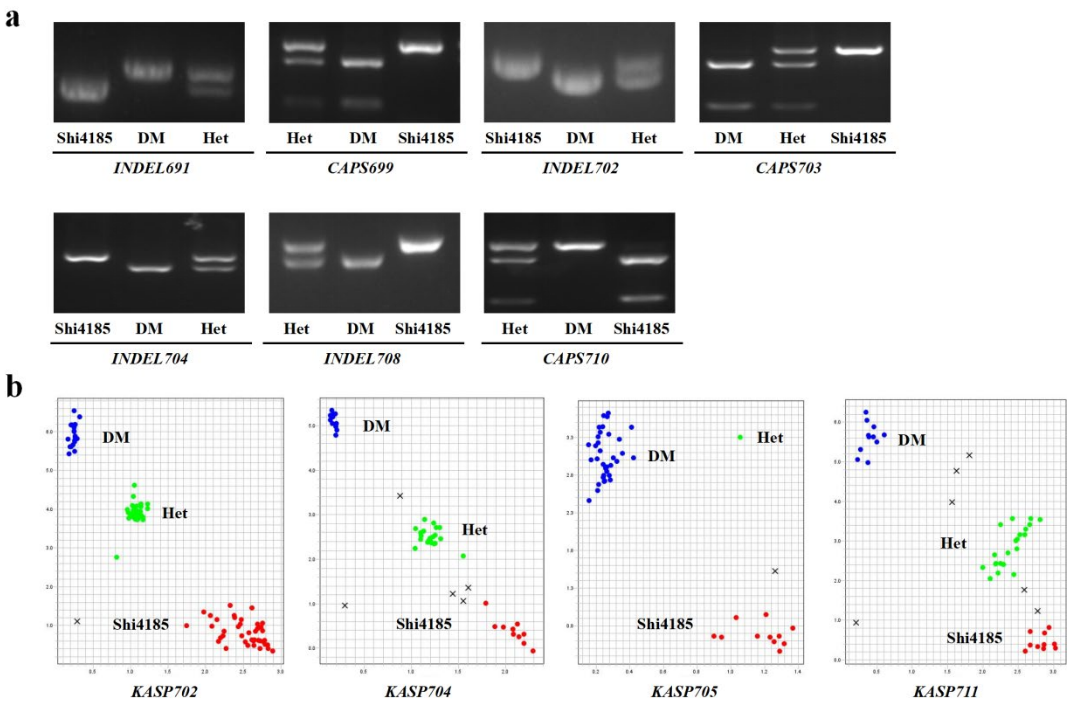


**Supplementary Figure 1.** Eleven PCR-based markers in mapping interval of *QSL.caas-6BL.1*. **a.** Agarose gel electrophoresis validation of INDEL and CAPS markers. The long-spike parent (Shi4185) and short-spike parent (DM) exhibit clear polymorphisms for both the (e.g., *InDel691*) and (e.g., *CAPS699*) markers, demonstrating their utility for haplotype discrimination. **b**. Genotyping of the ($F_{4:5}$) population using the Kompetitive Allele-Specific PCR (KASP) marker (e.g., *KASP702*). The scatter plot shows clear clustering of the three genotypic classes (blue: homozygous Shi4185, red: homozygous DM, green: heterozygous), which are tightly co-segregated with spike length phenotypes. This confirms the linkage of this marker with the *QTL QSL.caas-6BL.1* and highlights its potential for efficient marker-assisted selection.
