# Supplementary material for: Fine mapping and candidate gene mining of major QTL QSL.caas-6BL.1 for spike length in bread wheat (Triticum aestivum L.)
Source: Front Plant Sci. 2026 Jan 22;16:1744596. doi: 10.3389/fpls.2025.1744596 (PMC12872863; doi:10.3389/fpls.2025.1744596)
Supplement: Supplementary file 2 [file Image2.pdf]

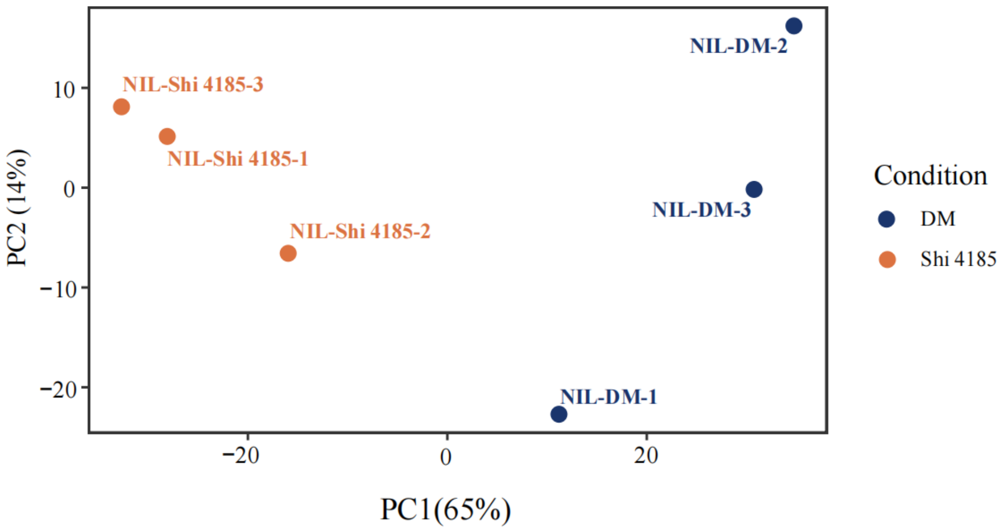

Supplementary Figure 2. Principal component analysis of the transcriptome. The plot, based on expression levels of all genes, shows tight clustering of biological replicates and clear separation of the two genotypes along the first principal component (PC1). PC1 and PC2 accounted for 65% and 14% of the total variance, respectively, demonstrating that genotype is the primary source of variation and confirming the high quality and reliability of the expression data.
