# Supplementary material for: Fine mapping and candidate gene mining of major QTL QSL.caas-6BL.1 for spike length in bread wheat (Triticum aestivum L.)
Source: Front Plant Sci. 2026 Jan 22;16:1744596. doi: 10.3389/fpls.2025.1744596 (PMC12872863; doi:10.3389/fpls.2025.1744596)
Supplement: Supplementary file 3 [file Image3.pdf]

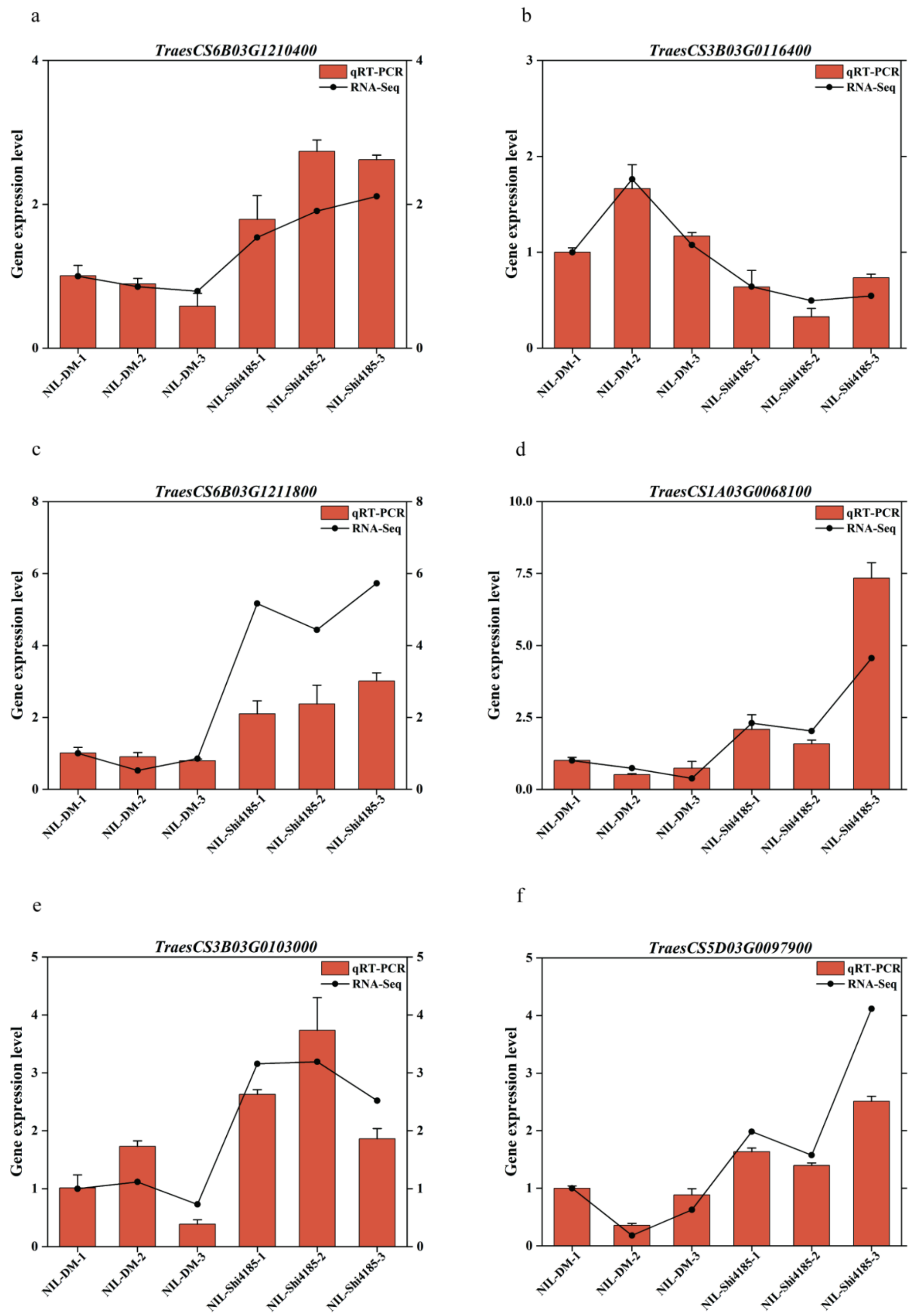

Supplementary Figure 3. The transcriptional profiles of selected genes were validated by quantitative real-time polymerase chain reaction (qRT-PCR). A comparison of gene expression levels from RNA-Seq data and qRT-PCR results for six selected genes is shown. The qRT-PCR analysis was performed with three biological replicates, using NIL-DM-1 as the calibrator sample for calculating the ΔΔCt values. The qRT-PCR results showed a highly significant positive correlation with the RNA-seq data.
