# Supplementary material for: Fine mapping and candidate gene mining of major QTL QSL.caas-6BL.1 for spike length in bread wheat (Triticum aestivum L.)
Source: Front Plant Sci. 2026 Jan 22;16:1744596. doi: 10.3389/fpls.2025.1744596 (PMC12872863; doi:10.3389/fpls.2025.1744596)
Supplement: Supplementary file 4 [file Image4.pdf]

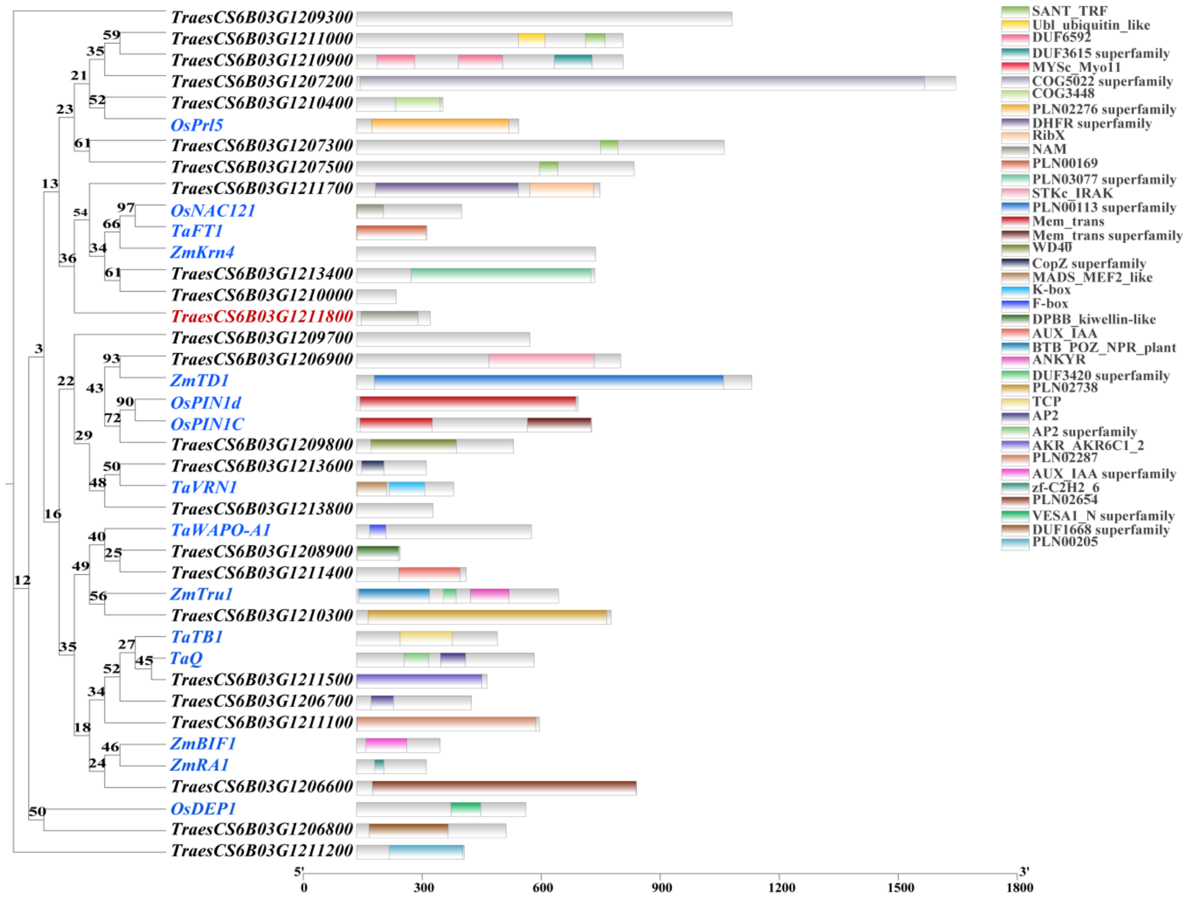

Supplementary Figure 4. Integrated phylogenetic and protein domain analysis of candidate genes within the *QSL.caas-6BL.1* interval with orthologs and known spike development genes. A maximum-likelihood phylogenetic tree was constructed using the protein sequences of the 25 high-confidence candidate genes from the fine-mapped *QSL.caas-6BL.1* interval, and a set of previously cloned spike development genes (labeled with blue, e.g., *OsNAC121*, *FT1*, *VRN1*, *Q*). Bootstrap support values from 1000 replicates are indicated at major nodes. The conserved protein domains for each corresponding protein, predicted by NCBI, are displayed as colored horizontal bars on the right of the tree. Key functional domains are annotated, such as the NAM domain present in the prime candidate *TraesCS6B03G1211800*. The candidate gene *TraesCS6B03G1211800* clusters closely with the known rice NAC transcription factor *OsNAC121*, supporting its potential role in spike development. The tree and domain architecture visualization were generated using TBtools (v2.363).
